# Logistics and CO_2_e emissions from beef cattle transportation in Brazil between 2018 and 2020

**DOI:** 10.1038/s41598-025-21931-5

**Published:** 2025-10-30

**Authors:** Fabio Martins Guerra Nunes Dias, Fredson Vieira e Silva, André Guimarães Maciel e Silva, Guilherme Jordão de Magalhães Rosa, José Bento Sterman Ferraz

**Affiliations:** 1 School of Animal Science and Food Engineering, University of São Paulo, São Paulo, SP Brazil; 2 Department of Agricultural Sciences, State University of Montes Claros, Janaúba Campus, Janaúba, MG Brazil; 3 Institute of Veterinary Medicine, Federal University of Pará, Castanhal Campus, Castanhal, PA Brazil; 4 Department of Animal and Dairy Sciences, University of Wisconsin-Madison, Madison, WI USA

**Keywords:** Climate-change mitigation, Environmental impact

## Abstract

This study analysed the logistics of beef cattle transportation in Brazil from 2018 to 2020, based on 288,267 freight orders involving over 19 million animals from 42,805 farms and 38 slaughterhouses in ten states. The analysis included vehicle categorization, travel distances, carcass weights, available space per animal, and CO_2_ equivalent (CO2e) emissions, which amounted to approximately 87,000 tonnes across 244,394 valid records. We hypothesized that emissions would vary substantially by truck class and transport conditions. Given that the median one-way transport distance was 160 km, all emission estimates were calculated for a 320 km round trip to reflect the full journey. Small trucks (up to 18 animals) were the most frequently used but also the least efficient; emissions for this class reached a median of 56.89 kg per ton of carcass and 13.99 kg per animal, with 75th percentiles as high as 98 kg/ton. In contrast, the Trailer54 class showed the highest efficiency, with a median of 37.77 kg/ton of carcass (IQR: 20.5–62.5). Across all classes, the wide gaps between the 25th and 75th percentiles point to substantial room for optimization. Emission intensity also varied between and within states, with median values per animal ranging from 8.74 kg in Minas Gerais to 17.31 kg in Rondônia. Intra-state variability was also considerable, as indicated by the wide interquartile ranges. Although emissions from livestock transport represent a small fraction of Brazil’s total GHG inventory, their spatial variability and operational inefficiencies make them strategically relevant for climate and logistics policy. These findings underscore the importance of region-specific strategies, fleet modernization, and improved logistical planning to support low-carbon beef supply chains.

**Supplementary Information:**

The online version contains supplementary material available at 10.1038/s41598-025-21931-5.

## Introduction

Global beef production reached 59.2 million tons in 2023, with the United States contributing 20.6% of this total, followed by Brazil with 17.9%^1^. In 2022, Brazil’s cattle herd totaled 202.78 million head, 42.31 million of which were slaughtered. Beef production amounted to 10.79 million tons, of which 7.78 million were destined for the domestic market and 3.02 million were exported^2^.

Given Brazil’s continental size, heterogeneous livestock systems^3^, and an extensive road network of approximately 1.7 million kilometers^4^, understanding how live cattle are transported to slaughterhouses is essential for improving logistical efficiency and reducing environmental impacts. Road transport is the dominant mode used, and since 2000, CO₂ emissions from heavy-duty vehicles have increased by more than 30%, with trucks accounting for 80% of this growth^5^.

In 2019, global greenhouse gas (GHG) emissions were estimated at 59 ± 6.6 Gt CO_2_e, with the transport sector responsible for approximately 15% of this total^6^. Brazil’s net emissions reached 2,039 Mt CO_2_e in 2022, equivalent to around 3.5% of global emissions^7^. Under its Nationally Determined Contribution (NDC), Brazil commits to reduce net GHG emissions to 1.20 Gt CO_2_e by 2030, equivalent to a 53.1% cut from 2005 levels, and to 1.05 Gt CO_2_e by 2035 (59% below 2005 levels)^8^. However, while most mitigation strategies focus on land use change, enteric methane, and energy consumption^9,10^ remain underrepresented in national inventories and poorly integrated into formal climate policies.

Recent studies have emphasized the strategic importance of transport-related emissions. Even when relatively small in climate accounting terms, emissions from transport logistics significantly affect supply chain traceability, regulatory compliance, and market access^11,12^. In Latin America, where beef supply chains operate with varied infrastructure and limited standardization, overlooking logistical emissions weakens governance and sustainability efforts^13,14^. In Brazil, cattle transport is primarily road-based, managed by meat processors, and involves a wide diversity of vehicle types, distances, and operating conditions, yet emissions from these operations remain largely unmonitored^10,15^.

Despite progress, such as the National Inventory of Atmospheric Emissions by Road Motor Vehicles^15^ and welfare-focused transport guidelines from the Ministry of Agriculture, Livestock, and Supply (MAPA)^16^, environmental efficiency criteria are still absent from animal transport regulation. Although some empirical studies conducted in Brazil have explored the influence of logistical decisions and infrastructure on environmental outcomes in agri-food systems^13,14^, they have not specifically addressed CO₂e emissions from cattle transport at a national scale.

In response to this gap, this study offers an empirical analysis of CO₂e emissions from cattle transport in Brazil, providing a detailed evidence base to support mitigation strategies, logistics optimization, and policy alignment with national and international climate commitments.

## Results

### Vehicle classification

The analysis of vehicles used in cattle transportation resulted in the classification into four distinct classes, combining two types of traction and three load capacities (Table 1). These classes were identified as Trailer36 and Trailer54 for trailers with average capacities of 36 and 54 animals, respectively, and Truck18 and Truck36 for trucks with average capacities of 18 and 36 animals, respectively. Additionally, freights involving more than one type of truck were categorized as Mixed.

**Table 1:** Vehicle classification for beef cattle transport considering traction (trailers or rigid trucks) and capacity (18, 19 to 36, and 37 to 54 heads).

Truck class (Nf = total freight orders, AMY = average manufacturing year, Nc = number of cattle transported)	Top truck type combinations in each class	Number of freight orders
Mixed (Nf = 43,873, Nc = 6,722,321)	Truck up to 11.50 m (18 hd) and double-deck trailer (40 hd)	8,146
	Double-deck trailer (54 hd) and truck up to 11.50 m (18 hd)	5,504
	Truck up to 11.50 m (18 hd) and trailer (27 hd)	5,483
	Trailer (27 hd) and double-dec k trailer (40 hd)	3,020
	Double-deck trailer (54 hd) and trailer (27 hd)	2,419
Trailer36 (Nf = 23,775, AMY = 2007, Nc = 916,680)	Trailer (27 hd)	14,152
	Trailer (22 hd)	6,493
	Trailer (30 hd)	2,243
Trailer54 (Nf = 43,497, AMY = 2012, Nc = 4,468,815)	Double-deck trailer (40 hd)	20,014
	Double-deck trailer (54 hd)	17,381
	Double-deck trailer (40 hd) and double-deck trailer (54 hd)	5,796
Truck18 (Nf = 173,587, AMY = 1999, Nc = 6,875,101)	Truck up to 11.50 m (18 hd)	145,715
	All-wheel drive truck up to 11.50 m (18 hd)	7,499
	ruck up to 11.50 m (18 hd) and all-wheel drive truck up to 11.50 m (18 hd)	7,303
Truck36 (Nf = 3,535, AMY = 2007, Nc = 114,585)	Single-axle tractor	3,532

Mixed, more than one truck type per freight order. Trailer36, trailers with an average capacity of 36 animals. Trailer54, trailers with an average load capacity of 54 animals. Truck18, rigid trucks with an average capacity of 18 animals. Truck36, rigid trucks with an average capacity of 36 animals.

The class capacities were determined on the basis of an average live weight between 510 and 570 kg, which is representative of the average beef male sent to slaughterhouses in Brazil. Freight operators naturally make adjustments when transporting lighter or heavier animals. Therefore, the capacities assigned to the class names pertain to average finished beef bulls.

The average manufacturing year for Trailer36 and Truck36 was 2007. For Trailer54 and Truck18, it was 2012 and 1999, respectively.

### Freight order distribution

The Truck18 class was the most prevalent class in freight orders, representing 60.2% of the total, followed by Trailer54 and Mixed (Table 2). The states of Acre, Bahia, Pará, Rondônia, and Tocantins predominantly used the Truck18 class in freight orders. Except for Goiás, which had a higher percentage of freight orders in the Mixed class, the other states also had a higher proportion of Truck18. Considering all the data, the Truck36 class was the least common class. Considering only Mixed freight orders, 73.7% of them involved a combination with Trailer54.

**Table 2:** Distribution of the percentage of beef cattle freight orders by truck class within each state.

Truck class	State										Overall
	AC	BA	GO	MG	MS	MT	PA	RO	SP	TO	
Mixed	0.2	7.8	31.5	16.5	16.6	16.5	9.1	5.7	23.0	12.4	15.2
Trailer36	0.0	1.0	13.2	28.9	9.8	6.0	3.5	1.4	11.4	1.0	8.2
Trailer54	0.2	11.3	24.7	3.9	23.0	19.6	2.2	2.2	30.6	8.9	15.1
Truck18	99.6	79.9	27.8	49.6	46.5	57.8	85.2	90.7	35.0	77.7	60.2
Truck36	0.0	0.0	2.8	1.1	4.1	0.1	0.0	0.0	0.0	0.0	1.2
Total	100	100	100	100	100	100	100	100	100	100	100

AC, Acre. BA, Bahia. GO, Goiás. MG, Minas Gerais. MS, Mato Grosso do Sul. MT, Mato Grosso. PA, Pará. RO, Rondônia. SP, São Paulo, TO, Tocantins. Mixed, more than one truck type per freight order. Trailer36, trailers with an average load capacity of 36 animals. Trailer54, trailers with an average load capacity of 54 animals. Truck18, rigid trucks with an average load capacity of 18 animals. Truck36, rigid trucks with an average load capacity of 36 animals.

The states of Mato Grosso, Mato Grosso do Sul, and Rondônia had the highest overall percentages of freight orders (Table 3). Specifically, within the Trailer36 class, Minas Gerais and Mato Grosso do Sul recorded the highest percentages of freight orders. For the Trailer54 class, Mato Grosso and Mato Grosso do Sul presented the highest percentages, whereas the Truck18 class presented the highest percentages in Mato Grosso and Rondônia. In the Truck36 class, Mato Grosso do Sul accounted for the highest percentage of freight orders.

**Table 3:** Distribution of the percentage of beef cattle freight orders by state within each truck class.

Truck class	State										Total
	AC	BA	GO	MG	MS	MT	PA	RO	SP	TO	
Mixed	0.0	1.0	18.6	8.2	22.9	28.4	5.8	5.5	8.4	1.2	100
Trailer36	0.0	0.1	14.4	26.7	25.1	19.0	4.3	2.5	7.7	0.2	100
Trailer54	0.0	1.5	14.7	2.0	32.2	33.9	1.5	2.1	11.3	0.8	100
Truck18	4.4	2.7	4.1	6.3	16.3	25.1	14.0	22.1	3.2	1.8	100
Truck36	0.0	0.0	20.3	6.6	71.0	2.1	0.0	0.0	0.0	0.0	100
Overall	2.6	2.0	9.0	7.6	21.1	26.1	9.9	14.7	5.6	1.4	100

AC, Acre. BA, Bahia. GO, Goiás. MG, Minas Gerais. MS, Mato Grosso do Sul. MT, Mato Grosso. PA, Pará. RO, Rondônia. SP, São Paulo, TO, Tocantins. Mixed, more than one truck type per freight order. Trailer36, trailers with an average load capacity of 36 animals. Trailer54, trailers with an average load capacity of 54 animals. Truck18, rigid trucks with an average load capacity of 18 animals. Truck36, rigid trucks with an average load capacity of 36 animals.

### Number of animals transported by truck class

A total of 6.9 million heads of cattle were transported in the Truck18 class, followed by 6.7 million in the Mixed class and 4.5 million in the Trailer54 class. The Trailer36 and Truck36 classes transported 0.9 million and 0.1 million heads of cattle, respectively. The Truck18 and Trailer54 classes presented the smallest and largest variations in the number of animals transported, respectively (Supplementary Table S1), excluding the Mixed class. Goiás and São Paulo are the states that transported the highest median number of cattle per vehicle, regardless of vehicle class.

More than 62% of the cattle in the states of Goiás, Mato Grosso do Sul, Mato Grosso, and São Paulo were transported via Trailer54 and Mixed classes (Table 4). In the states of Acre, Bahia, Pará, Rondônia, and Tocantins, most animals are transported via the Truck18 class.

**Table 4:** Distribution of the percentage of beef cattle heads transported by truck class within each state.

Truck class	State										Overall
	AC	BA	GO	MG	MS	MT	PA	RO	SP	TO	
Mixed	0.6	19.6	57.1	39.8	30.3	37.7	27.3	25.0	36.6	25.4	35.2
Trailer36	0.0	0.8	5.6	22.2	5.1	3.4	2.9	0.9	5.9	0.5	4.8
Trailer54	0.2	14.7	27.7	6.8	32.5	31.0	2.4	3.2	39.3	11.4	23.4
Truck18	99.2	64.9	8.9	30.6	30.2	27.9	67.5	70.9	18.2	62.7	36.0
Truck36	0.0	0.0	0.7	0.6	1.9	0.0	0.0	0.0	0.0	0.0	0.6
Total	100	100	100	100	100	100	100	100	100	100	100

AC, Acre. BA, Bahia. GO, Goiás. MG, Minas Gerais. MS, Mato Grosso do Sul. MT, Mato Grosso. PA, Pará. RO, Rondônia. SP, São Paulo, TO, Tocantins. Mixed, more than one truck type per freight order. Trailer36, trailers with an average load capacity of 36 animals. Trailer54, trailers with an average load capacity of 54 animals. Truck18, rigid trucks with an average load capacity of 18 animals. Truck36, rigid trucks with an average load capacity of 36 animals.

### Distances travelled by vehicles from farms to slaughterhouses

The data on the distance from the farm to the slaughterhouse, considering all truck classes, revealed the following percentiles: 10th percentile at 50 km, 25th percentile at 89 km, median at 160 km, 75th percentile at 267 km, and 90th percentile at 393 km. Compared with the other classes, the Trailer54 truck class covered the longest distances (median of 196 km) (Supplementary Table S2). The other truck classes travelled distances close to the overall median. The longest distances were observed in the states of Goiás, Mato Grosso do Sul, Rondônia, and Bahia.

### Median carcass weights by truck class and state

Overall, the median carcass weights for females and males were 217 and 293 kg, respectively. The median carcass weight for female cattle transported in Trailer54 and Mixed classes was among the highest, at 223 and 228 kg, respectively (Supplementary Table S3), whereas males presented similar trends for the same classes, at 298 and 303 kg (Supplementary Table S4). In contrast, animals transported by the Truck18 class consistently had the lightest median carcasses for both females and males. The states of Goiás and Mato Grosso had the heaviest carcasses for both sexes, whereas Rondônia had the lightest carcasses.

### Space available per animal during transportation

On the basis of the median linear space per animal across different truck classes, the Trailer54 class provided the most space for all categories (0.6 m/animal), offering similar conditions for males and females (Supplementary Table S5). The Trailer36 class also provided relatively more space for these categories. The Truck18 class consistently provided the least space (0.48 and 0.54 m for females and males, respectively), whereas the Truck36 class offered slightly more space than did Truck18 but less space than did the Trailer36 class.

### GHG emission estimates by truck class and state

The total estimated CO₂e emissions amounted to 86,946.63 tonnes, based on 244,394 freight orders. Mixed-class shipments, 43,873 freight orders involving combinations of two or more truck types, were excluded to ensure methodological consistency, as their heterogeneity prevented accurate allocation of emissions by vehicle class. As a result, the total reported emissions are underestimated.

**Fig. 1:**
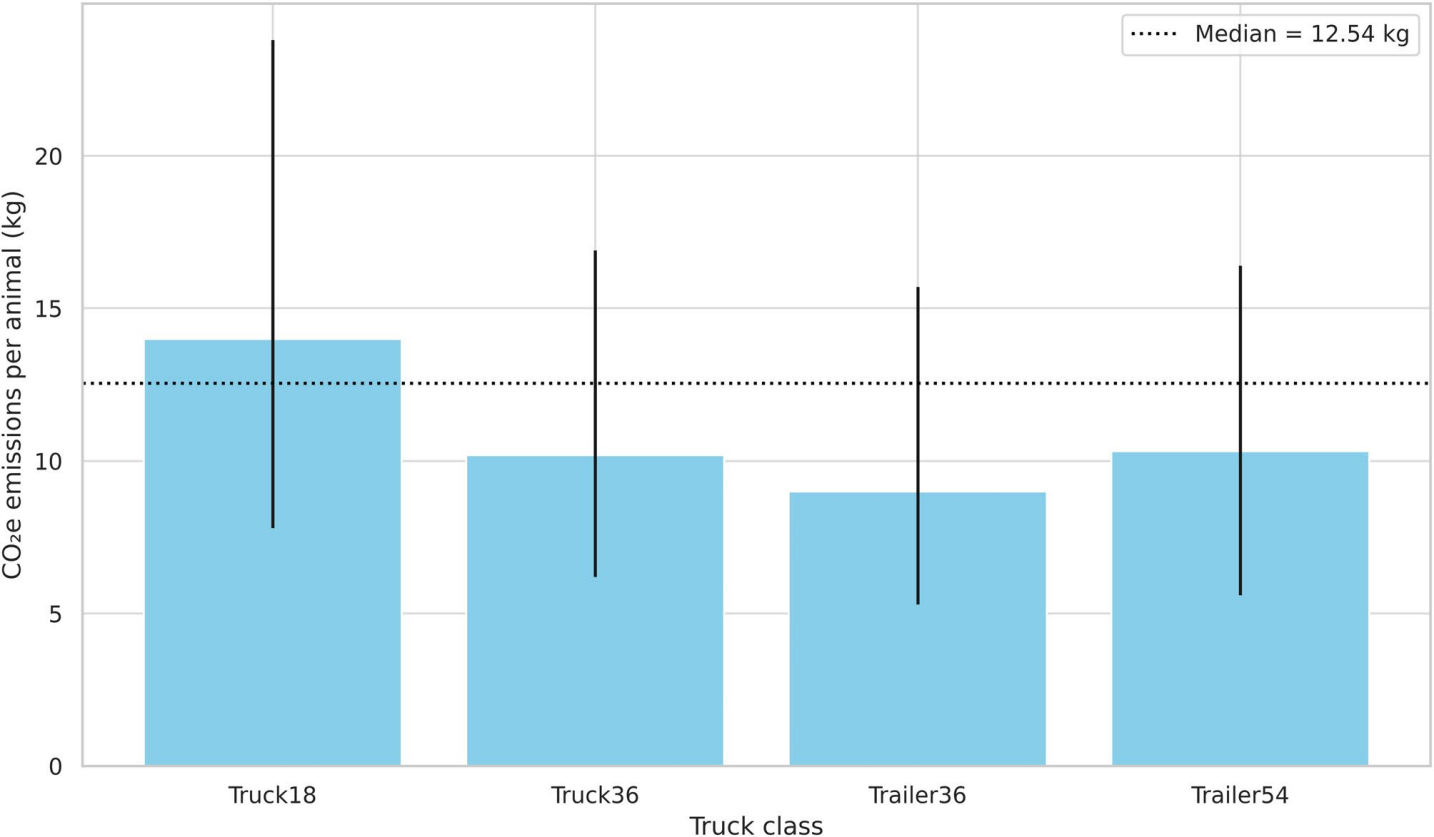
Median estimates and interquartile range (25th and 75th percentiles) of CO₂ equivalent (CO₂e) emissions per animal by truck class, based on a 320 km round trip. Bars represent median values; vertical lines indicate the 25th and 75th percentiles. The dashed horizontal line represents the overall median across all truck classes.

**Fig. 2:**
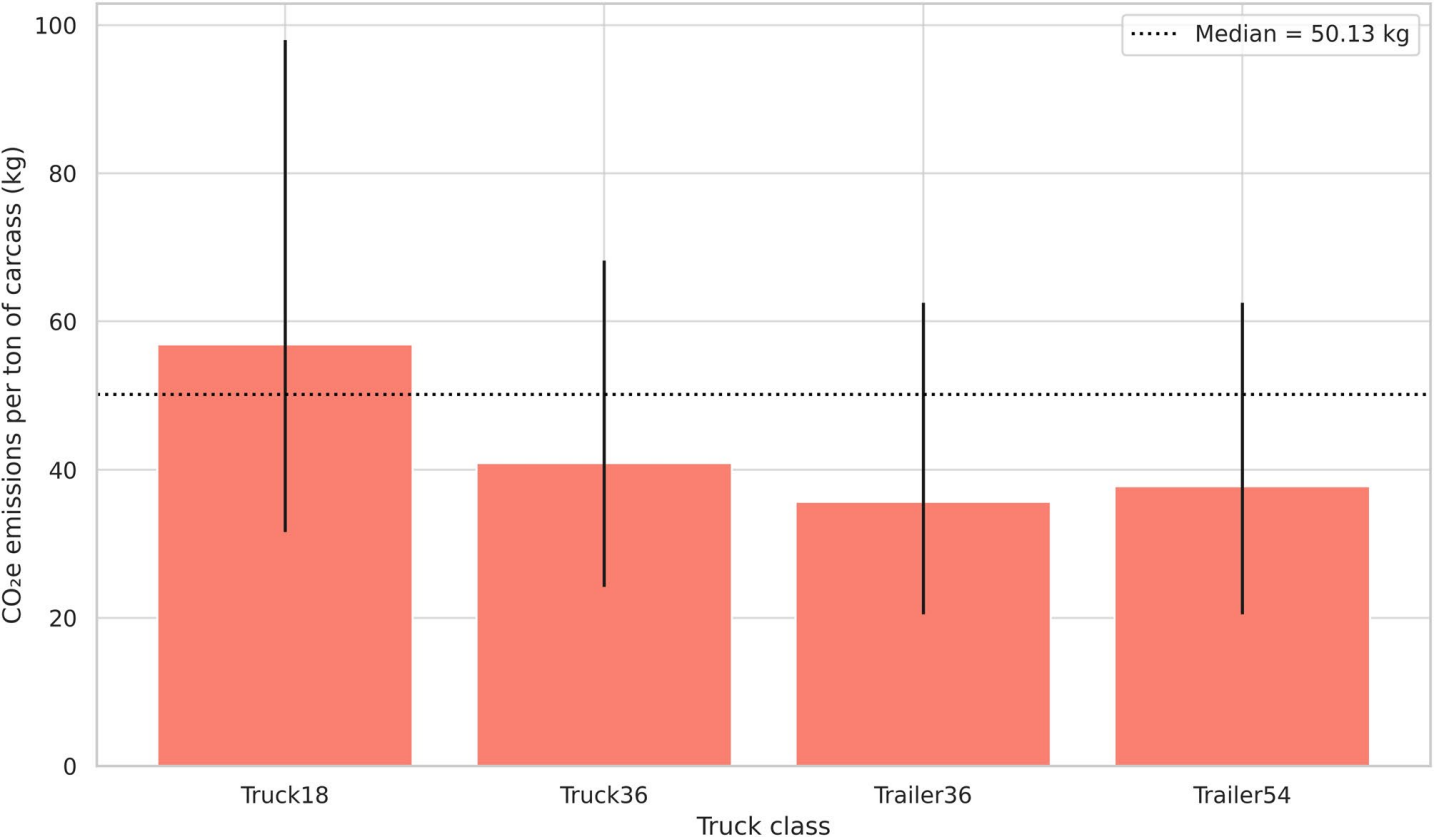
Median estimates and interquartile range (25th and 75th percentiles) of CO₂ equivalent (CO₂e) emissions per ton of carcass l by truck class, based on a 320 km round trip. Bars represent median values; vertical lines indicate the 25th and 75th percentiles. The dashed horizontal line represents the overall median across all truck classes.

Trailer54 was classified as heavy (GCW > 40 tons), while the other truck classes were considered semiheavy. The overall median CO₂e emission per animal was 12.54 kg, with an interquartile range (IQR) of 7.0 to 21.4 kg (Fig. 1; Supplementary Table S6), and 50.13 kg per ton of carcass (IQR: 27.7 to 86.5 kg) (Fig. 2).

Truck18 exhibited the highest emissions, both per animal and per ton of carcass, reaching up to 23.8 kg per animal and 98 kg per ton. Trailer36 had the lowest emissions, with median values of 9.00 kg (IQR: 5.3–15.7) per animal and 35.66 kg (IQR: 20.5–62.5) per ton. CO₂e emissions per kilometer travelled ranged from 27.34 to 46.49 g per animal. Per ton of carcass, values ranged from 96.83 to 187.2 g.

**Fig. 3:**
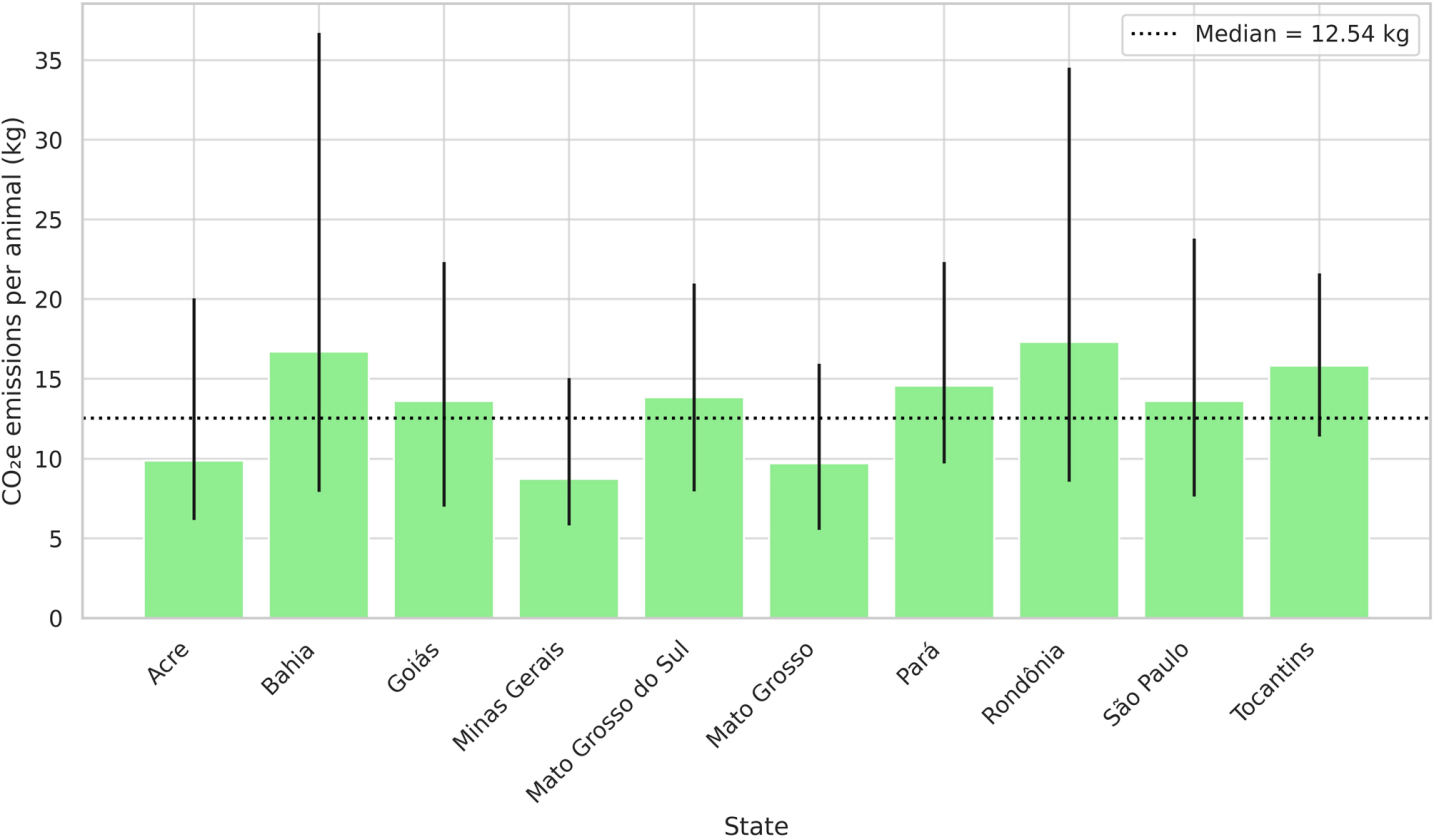
Median estimates and interquartile range (25th and 75th percentiles) of CO₂ equivalent (CO₂e) emissions per animal by state, based on a 320 km round trip. Bars represent median values; vertical lines indicate the 25th and 75th percentiles. The dashed horizontal line represents the overall median across all truck classes.

**Fig. 4:**
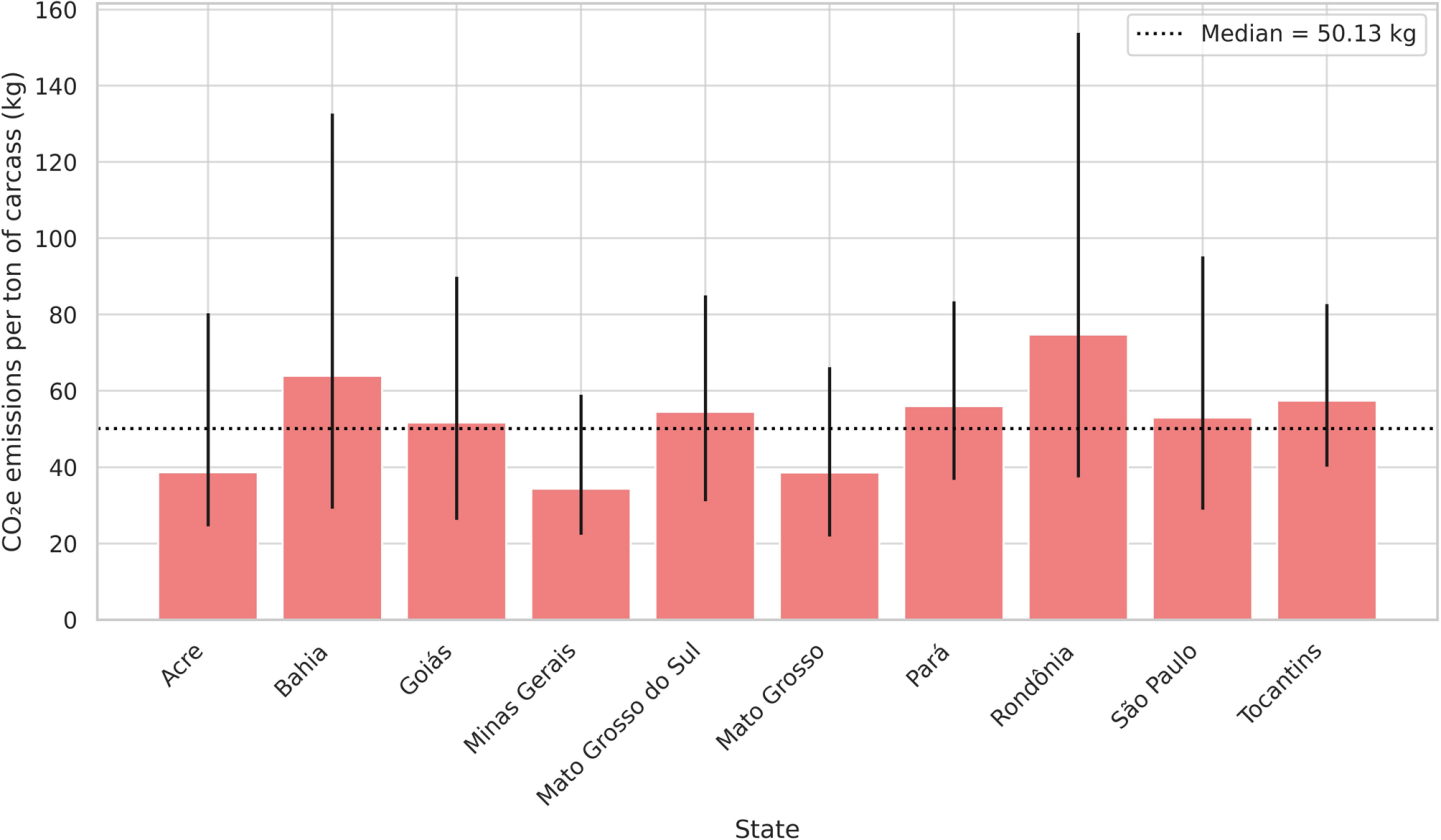
Median estimates and interquartile range (25th and 75th percentiles) of CO₂ equivalent (CO₂e) emissions per ton of carcass by state, based on a 320 km round trip. Bars represent median values; vertical lines indicate the 25th and 75th percentiles. The dashed horizontal line represents the overall median across all truck classes.

At the state level, Minas Gerais had the lowest emissions per animal, with a median of 8.74 kg (IQR: 5.83–15.05), whereas Rondônia had the highest, at 17.31 kg (IQR: 8.57–34.53) (Fig. 3; Supplementary Table S7). For each ton of carcass, values ranged from 34.40 to 74.73 kg (Fig. 4). Below the national median are Minas Gerais, Mato Grosso, Acre, and São Paulo; above are Bahia, Goiás, Mato Grosso do Sul, Pará, Rondônia, and Tocantins.

## Discussion

The detailed analysis of beef cattle transportation in Brazil provides critical insights into its logistics and environmental impact. This study provides a comprehensive overview of cattle transportation practices, highlights significant regional variations and identifies priority areas for improvement.

### Truck classification and operational usage patterns

The categorization of vehicles into classes by combining traction types and load capacity allowed for a clearer understanding of the predominant practices in cattle transportation. The Truck18 class was the most commonly used class, especially in the states of Acre, Bahia, Pará, Rondônia, and Tocantins, representing 60% of total freight orders but only 36% of the number of animals transported. The rigid truck classes, Truck18 and Truck36, transported a greater percentage of cows compared to steers and beef bulls, with 50% and 48% of the cows and 41% and 43% of the males, respectively. This finding is possibly due to the number of cull cows from both beef and dairy herds^17^. Moreover, culled animals typically come from smaller herds and farms, as breeding activities are often associated with small producers, resulting in smaller lot sizes for transportation. Cull dairy cows, in particular, have been shown to be transported to slaughterhouses in smaller vehicles and over shorter distances than beef cattle^18^.

Conversely, states such as Goiás, Mato Grosso, Mato Grosso do Sul, and São Paulo showed greater use of Trailer54 and Mixed classes, reflecting the presence of larger farms and/or large feedlots, as all these states have large cattle populations^19^. The high proportion of Trailer54 in the Mixed freight orders indicates an adjustment in the total quantity of cattle to better fit truck size capacities by using a combination of the Trailer54 class and one or more smaller trucks. The states of Goiás, Mato Grosso, Mato Grosso do Sul, and São Paulo transported 63% of the total number of cattle in large trucks.

The Trailer36 and Trailer54 classes transported a lower percentage of cull cows compared to steers and beef bulls, with 44% and 31% of the cows and 48% and 59% of the males, respectively. However, the preference for smaller trucks in certain states (Acre, Bahia, Pará, Rondônia, and Tocantins) may reflect logistical or economic constraints, such as roads and farm access conditions.

### Impact of truck class on carcass weight and transport distance

This study highlighted a relationship between vehicle class and carcass weight. Larger capacity vehicles (Trailer54 and Mixed classes) transported animals with higher median weights, indicating that properties finishing a greater number of animals at a time (i.e., large freight orders) produce more males, which have higher final weights^20^. The distances travelled varied significantly between vehicle classes and states. The Trailer54 class stood out for covering the longest distances (Supplementary Table S2). The analysis revealed that the median distance travelled was 160 km, with significant variations between states, indicating the need for regionally adapted strategies. The deciles and quartiles of the distances travelled by these vehicles can be used to establish Brazilian standards for cattle transportation from farms to industries, as, to our knowledge, no such reference currently exists in Brazil. One of the most important concerns regarding welfare during transport includes the total duration of the journey^21,22^. Thus, on the basis of the distances travelled by vehicles from farms to slaughterhouses, the following classification is suggested for beef cattle transportation in Brazil: ‘very short’ for transports up to 50 km, ‘short’ for transports up to 90 km, ‘medium’ for transports up to 160 km, ‘medium–long’ for transports up to 270 km, ‘long’ for transports up to 399 km, and ‘very long’ for transports from 400 km onwards.

### Fleet efficiency and CO2e emission reduction strategies

The Trailer36 class showed the highest efficiency in terms of CO₂e emissions, partly because of the shorter distances travelled. Compared with the other classes, the Trailer54 class registered slightly higher emissions, even covering approximately 95 km more in round trips (Supplementary Table S2). The efficiency of the Trailer54 class became evident when analysing emissions per kilometre travelled, whereas the inefficiency of the Truck18 class was also evident, emitting 93.33% more CO₂e than the Trailer54 class per ton of carcass delivered.

Another relevant point is the age of the trucks^23^. A comparison of the fuel efficiency of semiheavy trucks manufactured in 1999 with those from 2007 revealed that there was a 9.3% improvement, directly impacting CO_2_e emissions^24^. Although the Trailer54 class has a 28.3% lower fuel efficiency compared to Trailer36 and Truck36, its greater load capacity results in lower emissions per ton transported. This makes it the most efficient class when considering total CO₂e emissions per unit of carcass. Therefore, modernizing the fleet with a focus on energy efficiency and replacing older trucks with newer, higher-capacity models such as the Trailer54 class can significantly reduce CO_2_e emissions per animal or ton of carcass delivered.

Even within the same truck class, CO_2_e emissions per round-trip journey varied significantly between the 25th and 75th percentiles, highlighting a potential for mitigation within the category itself. For example, in the Truck18 class, emissions per animal ranged from 7.8 kg (25th percentile) to 23.8 kg (75th percentile), a 205% increase. In contrast, the more efficient Trailer54 class showed a narrower range, from 5.6 to 16.4 kg (193%). These values refer to total emissions per animal over a full round trip and reflect operational conditions observed in real freight data. The text above shows that the number of animals per load, as well as the truck type, can influence CO₂e emissions. This result is in line with the findings of Duarte et al. (2019)^25^, who reported that higher freight volumes significantly reduce environmental impacts over long distances in fresh food logistics.

While emissions per kilometer are standardized, their overall impact is directly proportional to the distance traveled. For instance, in the Truck18 class, which emits 46.49 g CO₂e per animal per kilometer, a 25th percentile journey of 89 km generates approximately 4.1 kg per animal, while a 75th percentile journey of 267 km leads to 12.4 kg — a 202% increase. For the Trailer54 class, with 27.34 g per km, the same distances yield 2.4 kg and 7.3 kg, respectively. These examples show that even efficient trucks accumulate significant emissions on longer routes, or promoting slaughter in geographically closer facilities, especially in states with a high share of inefficient transportation.

Among the few studies that isolate transport emissions, Castillo^11^ reported a value of 0.05533 kg CO₂e per ton per kilometre for cattle transport in Colombia, equivalent to 17.7 kg CO_2_e per ton over a 320 km journey. This value aligns with the lower range of our results, particularly in states and vehicle classes with more efficient logistical performance, reinforcing the plausibility of the estimates obtained in the Brazilian context.

While most international life cycle assessments report total emissions from beef systems—including feed production, enteric fermentation, and processing—transport is typically estimated to account for about 5% of total GHG emissions. Based on this proportion, transport-related emissions would range from approximately 800 to 1,370 kg CO_2_e per ton of carcass, given total emissions of 16 to 27 kg CO_2_e per kg of meat reported in studies from Australia, Europe, and Canada^26–28^. Although not directly comparable due to differences in system boundaries, these figures confirm that our results—ranging from 35 to 98 kg CO_2_e per ton—are within a plausible range for transport-only estimates and highlight Brazil’s logistical heterogeneity and structural challenges.

We conducted a simulation based on a 534 km round trip (corresponding to the 75th percentile of the distances observed) and proportionally adjusted the previously calculated emission values for the standard 320 km trip. In this longer-distance scenario, the most inefficient case — Truck18 (75th percentile) — reached 39.38 kg CO_2_e per animal, while a more efficient case — Trailer54 (25th percentile) — emitted only 9.35 kg. Considering the 42.31 million cattle slaughtered in 2022, these scenarios would result in 1.67 million tons of CO₂e in the less efficient case (0.0819% of Brazil’s net emissions in 2022) and 396 thousand tons in the most efficient one (0.0194%). The difference between them represents a potential savings of 1.27 million tons or 0.0623% of the country’s annual emissions, highlighting the mitigation potential of fleet modernization and the strategic allocation of more efficient trucks to long-distance routes.

However, CO_2_e emission levels in diesel trucks remain high^10^. Furthermore, in regions with poor road conditions, larger trucks, such as those in the Trailer54 class, may be unable to access certain farms because of inadequate road infrastructure. This forces the use of smaller trucks, such as the Truck18 class, which can navigate these rough terrains but result in higher CO₂e emissions per unit of cattle transported. This study considered only CO₂e emissions without considering factors such as whether the truck operated at full load capacity during a round trip, which could have altered the results^29^.

In addition to optimizing truck age and class, optimizing the transported load is a key strategy to mitigate the environmental impact of beef cattle transportation. Underutilizing truck capacity, particularly in the Trailer54 class, often results in increased CO_2_e emissions per ton of carcass. This inefficiency is more pronounced when the lower quartile of animals transported per truck class is analysed (Supplementary Tables S1 and S5). Adopting sustainable technologies and ensuring full truckloads are essential steps to reduce emissions. Therefore, improvements in logistics planning meaningfully reduce GHG emissions, which enhances overall efficiency.

It is worth noting that the emission estimates presented in this study are based on CO₂-equivalent values derived from the IPCC Tier 2 methodology, which assumes complete diesel combustion. Therefore, this approach does not capture other air pollutants, such as particulate matter or nitrogen oxides, that are commonly associated with human health risks. These substances have been linked to respiratory and cardiovascular diseases, particularly in populations chronically exposed to vehicle emissions^30,31^. While this was beyond the scope of our analysis, we highlight the importance of incorporating such health-related dimensions in future evaluations of livestock transport systems.

While it is important to standardize load limits according to vehicle class and animal weight^32,33^, the analysis of the space available per animal during transportation suggests that, in general, considering the median, the space provided was adequate according to the recommendations of the Ministry of Agriculture, Livestock, and Supply. However, additional analysis is needed because of the variations observed when evaluating the 25th and 75th percentiles. Therefore, monitoring and adjustments are essential when necessary to ensure optimal conditions and prevent animal welfare issues, such as injuries or mortality during transport^34,35^.

Compliance with the regulations of the Brazilian Ministry of Agriculture, Livestock, and Supply^36^ is essential for ensuring animal welfare^37^ and meat quality^38^, which are critical factors for sustainability and acceptance in the global market. Although the proposed name for the truck classes indicates the average maximum number of animals transported, it is important to use it cautiously, as this variation depends on factors previously discussed, such as weight and sex categories.

Figures 3 and 4 shows significant variation in CO₂e emissions related to cattle transportation across Brazilian states. The relative inefficiency observed in the states of Rondônia, Pará, Bahia, and Tocantins can be attributed to the greater use of the Truck18 class, in addition to longer distances and lighter carcasses (Supplementary Tables S3 and S4). Although over 99% of freight orders in Acre used the Truck18 class, its emission intensity remained below the overall median. This is mainly due to its shorter transport distances—the lowest among all states—highlighting the multidimensional nature of emission outcomes. Minas Gerais, the state with the lowest emissions, consistently performed better than the national median across all key variables.

Thus, each Brazilian state requires specific policies to improve animal transport logistics and reduce the environmental impact per animal transported, especially in states facing efficiency challenges. Therefore, our results reveal substantial spatial variability in emission efficiency across geographic regions, which aligns with findings from other agricultural supply chains. For instance, the carbon footprint of Brazilian soy exports varied up to sixfold depending on the municipality of origin, highlighting the importance of spatial resolution in emission assessments^39^.

Intra-state variability in CO_2_e emissions was also considerable and deserves attention. Even within the same state, emission estimates showed wide dispersion, as indicated by the 25th and 75th percentiles. For example, in Rondônia, emissions per ton of carcass ranged from 37.38 to 153.93 kg, representing a fourfold difference. Similarly, in Bahia, values ranged from 29.15 to 132.72 kg/ton, while Goiás showed a narrower but still relevant spread (26.27 to 89.93 kg/ton). These disparities highlight the heterogeneity in operational conditions, infrastructure, and load optimisation even within state boundaries. Therefore, local dynamics must be considered when designing targeted mitigation strategies.

Although the specific road conditions for each journey were not recorded, the results presented here may not fully reflect the impact of deteriorated road infrastructure. According to the National Confederation of Transport^4^, the proportion of paved roads rated as regular, poor, or very poor reaches 97.8% in Acre, 72.7% in Mato Grosso, 66.7% in Tocantins, and over 59% in Bahia and Pará—states that also showed the highest CO_2_e emission intensities. Even in Goiás and Minas Gerais, which recorded the lowest emissions, a significant portion of the road network remains in suboptimal condition, above 53%. National simulations indicate that poor road quality can reduce fuel efficiency by up to 14.7%. Additionally, other unaccounted variables, such as eco-driving practices, may also influence emission levels^40^.

Although the CO₂e emissions reported in this study represent a small share of Brazil’s total greenhouse gas inventory, they contribute cumulatively to global warming. Elevated atmospheric CO₂ and other greenhouse gases are strongly linked to rising global temperatures, altered precipitation regimes, and more frequent extreme weather events, with adverse effects on agriculture and food systems^41–43^. In tropical regions like Brazil, these climate shifts can reduce pasture biomass and forage quality, intensify drought and heatwave occurrence, and increase livestock heat stress—factors linked to reduced animal welfare and productivity^44,45^. Such pressures may destabilize rural supply chains and threaten regional food security. Therefore, even relatively modest emission sources—such as transport logistics—should be considered within integrated climate mitigation and adaptation frameworks.

### Policy implications and recommendations

As the world’s second-largest beef producer, Brazil faces increasing pressure to meet climate commitments while maintaining productivity and market competitiveness. In this context, reducing emissions from transport logistics, particularly in road-based systems, represents a practical and underutilized opportunity for mitigation.

Our findings show that emissions vary not only between truck classes but also across and within states, revealing operational inefficiencies and infrastructure disparities that can be addressed through targeted policy interventions. While not explicitly addressed in this analysis, factors such as credit availability, regulatory incentives, and the high number of small-scale producers may help explain the logistical disparities observed and merit exploration in future research. Investing in rural road improvements, especially in key livestock-producing regions, can enable the use of more efficient truck classes and reduce total emissions. Similarly, tax incentives or subsidized credit for fleet modernization — prioritizing older vehicles operating in remote areas — would contribute to decarbonization without compromising supply chain flow.

Unlike most LCA models that apply generalized emission factors across entire systems, this study reveals considerable spatial and operational variability. These results suggest that the transport segment, though often treated as marginal in GHG calculations, may play a disproportionate role in traceability and regional inequality, challenging the narrative of uniform marginality.

Integrating CO_2_e monitoring into logistics platforms and promoting best practices in transport operations and coordination would enhance transparency and efficiency. These measures should be aligned with broader sustainability frameworks, including Brazil’s NDC goals and low-carbon agriculture programs. Furthermore, policies promoting traceability and environmental certification in livestock logistics could strengthen Brazil’s position in increasingly regulated international markets.

Importantly, the inclusion of transport-related emissions in national inventories and climate policies, still limited in scope, would allow for more comprehensive climate planning and reporting. While emissions from livestock transport are modest in magnitude, they offer didactic value and high replicability. Each sectoral improvement, however incremental, contributes to building a low-emission economy. The case of cattle transport exemplifies how localized, data-informed strategies can generate scalable lessons for agri-food logistics in Brazil and across Latin America.

Although optimizing logistics contributes to emission reductions, complementary strategies such as improving animal productivity, reducing slaughter age, and optimizing feed conversion rates remain essential to achieving broader mitigation goals in beef systems^14,46^.

## Methods

### Data description

**Fig. 5:**
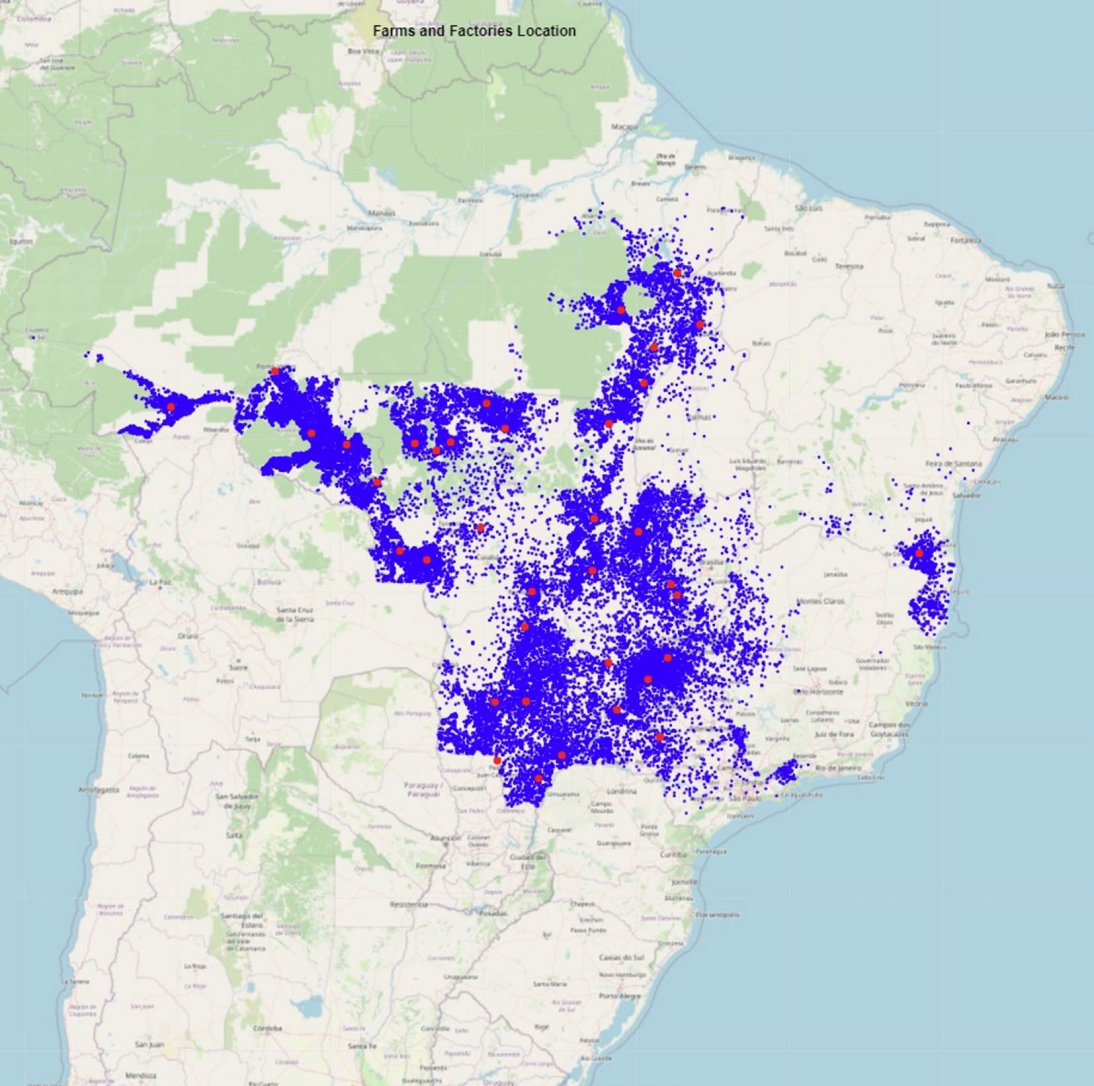
The dataset represents the geographical distribution of beef cattle transportation from 42,805 farms to 38 slaughterhouses across Brazil. Each blue point indicates a farm’s location, whereas the red points mark the slaughterhouses. The map was generated by the authors using the *folium* package (version 0.12.1, https://python-visualization.github.io/folium/) in Python 3.8.8 (https://www.python.org/), with original data collected during this study.

The dataset analysed covers three years of beef cattle slaughter in ten states (federative units) of Brazil out of a total of 26 states. It was provided by a major beef processing company operating across these ten states: Acre, Bahia, Goiás, Minas Gerais, Mato Grosso do Sul, Mato Grosso, Pará, Rondônia, São Paulo, and Tocantins. It contains detailed operational information on cattle transportation from 42,805 farms to 38 slaughterhouses (Fig. 5), including logistics and carcass characteristics. In Brazil, cattle transport from farms to slaughterhouses is usually managed by the meat industry, which enables the consolidation of large-scale, standardized datasets containing the origin of animals, vehicle types, carcass weights, and slaughter locations.

### Data analysis

For the data analysis, each freight order — representing a loading operation at a farm, regardless of the number of animals or trucks used — was considered the observational unit. A total of 301,653 freight orders issued between January 1, 2018, and December 31, 2020, were initially evaluated. Of these, 13,386 orders (4.44%) were excluded due to inconsistencies in transport distance, number of animals, or carcass weight. The final sample comprised 288,267 freight orders, involving 19,097,502 cattle. This figure corresponds to approximately 21.2% of all cattle slaughtered in Brazil during the same period^47^.

### Vehicle classification

The freight orders presented more than 600 types of vehicles or combinations of different vehicles in the transportation of larger lots. To provide an overview of cattle transportation from farms to slaughterhouses, the vehicles listed in the freight orders were grouped according to the type of traction and their load capacity, forming truck classes. The types of traction used were categorized as vehicles pulled by tractor units (trailers) or rigid trucks (trucks). In terms of load capacity, the vehicles were classified as having the capacity to transport an average of 18, 19 to 36, and 37 to 54 heads per load, considering the weight most common for finished males delivered (510–570 kg).

### Analysis of freight orders and carcass weights

The analysis included the distribution of freight orders within truck classes in each state, as well as the distribution of the number of cattle transported in each truck class by state. Additionally, carcass weights were analysed by truck class and state. Each freight order included the average carcass weight multiplied by the number of animals, which was divided by the number of trucks used on that day. Additionally, the dataset includes the distance travelled by the vehicles from the farms to the slaughterhouses.

### Space available per animal during transportation

To analyse the area available per animal within each type of truck, the main types of trucks used within each class and the quantities of animals transported were characterized. The characterization was performed by determining the number of compartments in each vehicle’s trailer and their dimensions. To ensure that the analysis included only trucks with known characteristics, the data were filtered to include only specific types of trucks, excluding those freight orders that involved more than one type of truck. The sample size for this analysis was 244,394 freight orders, corresponding to 85% of the total. The average number of animals transported per truck was calculated by dividing the total number of animals transported by the number of trucks in each freight order.

The live weight of the animals was calculated from the average carcass weight of each freight order, divided by 0.5 for females and 0.54 for males, representing the 50% and 54% average yields observed in Brazil for Zebu cattle and terminal crosses for females and males, respectively^48–50^. On the basis of the guidelines and regulations of the Brazilian Ministry of Agriculture, Livestock, and Supply, which establish the optimal linear space of vehicle trailer compartments according to the live weight of the animals^36^, the median space per animal was calculated for each vehicle class, considering sex. The linear dimensions of the truck compartments were specified to ensure the correct allocation of the animals. Brazilian legislation adopts linear measurements of the load compartment lengths to determine the number of animals per truck or trailer, considering that these vehicles have a standardized width of 2.6 m, the maximum limit for regular and public roads^51^.

### GHG emission estimates

The methodology for estimating GHG emissions generated by vehicle classes followed the guidelines of the Intergovernmental Panel on Climate Change^52^, which uses emission factors and energy efficiencies specific to trucks in Brazil to ensure greater accuracy in the estimates^53^. This approach aligns with a Tier 2 methodology, as the Brazilian National Inventory adjusts Tier 1 IPCC emission factors by incorporating fleet-specific parameters—particularly vehicle energy efficiency and year of manufacture. This correction allows the model to account for differences in fuel consumption and emissions across older and newer trucks, improving the accuracy of estimates. The dataset used in our study includes the manufacturing years of the vehicles, allowing for more detailed estimations.

The truck classes were initially reorganized into two main groups, semiheavy and heavy trucks, with weights below or equal to and above 40 tons, respectively^15,54^. This classification is based on the gross vehicle weight (GVW) or GCW. GVW refers to the total weight of the vehicle plus its load, which is used for rigid trucks, whereas GCW includes the combined weight of the tractor unit and trailer, which is applicable to articulated vehicles.

### Data editing and descriptive analysis

The raw data were obtained directly from the company responsible for cattle transport and slaughter, delivered in 72 standardized Excel spreadsheets containing information from all slaughter units for each fortnight. All records were automatically generated by the company’s enterprise resource planning (ERP) system, which is routinely used for transport scheduling and carcass management. No survey instruments or questionnaires were applied.

This ERP system ensures high-frequency, objective, and standardized data collection, enabling traceability across freight operations. These data were consolidated via the Pandas package in Python 3.8.8. Descriptive analyses were conducted via RStudio software^55^. Initially, the data were imported from the CSV file via the readxl package. Then, the data were manipulated and transformed via the dplyr package, which was used to group the data and calculate percentage distributions. Medians and quantiles (10th, 25th, 75th, and 90th) were calculated via native R functions and the dplyr package.

The median function was used to calculate the median, and the quantile function was used to calculate quartiles and deciles. For the statistical summary of the data, the summarize function from the dplyr package was employed. The percentage distribution tables were created via the tidyr package to reorganize the data. Percentages were calculated on the basis of the count of occurrences of each class within each state. Percentile distributions (25th, and 75th) were used as an exploratory sensitivity analysis to assess the variability in CO₂e emissions under different operational conditions.

## Supplementary Information

Below is the link to the electronic supplementary material.

Supplementary Material 1

## Data Availability

The data used in this study are available in the Figshare repository, accessible via the DOI: https://doi.org/10.6084/m9.figshare.28193201.v1 . The raw data are included in this repository, while the supplementary tables are in a separate file.
