# Supplementary material for: Logistics and CO2e emissions from beef cattle transportation in Brazil between 2018 and 2020
Source: Sci Rep. 2025 Oct 30;15:38063. doi: 10.1038/s41598-025-21931-5 (PMC12575759; doi:10.1038/s41598-025-21931-5)
Supplement: Supplementary file 1 — Supplementary Material 1 [file 41598_2025_21931_MOESM1_ESM.pdf]

Supplementary Table S1. Medians and 25th and 75th percentiles of the number of beef cattle transported by truck class and originating state

State	Truck class					Median
	Mixed	Trailer36	Trailer54	Truck18	Truck36	
AC	28 (25–30)		54 (52–54)	19 (18–20)		19 (18–20)
BA	30 (26–33)	30 (24–40)	40 (32–40)	20 (19–20)		20 (20–22)
GO	32 (25–38)	27 (25–27)	45 (40–50)	18 (18–18)	22 (22–27)	27 (18–40)
MG	22 (20–26)	26 (22–27)	40 (36–44)	18 (18–20)	27 (27–29)	20 (18–26)
MS	30 (23–35)	25 (21–30)	40 (37–46)	20 (20–21)	22 (20–24)	22 (20–34)
MT	31 (26–36)	26 (24–30)	40 (38–47)	18 (18–20)	24 (22–25)	20 (18–34)
PA	25 (22–30)	27 (26–30)	40 (36–55)	18 (18–18)		18 (18–20)
RO	28 (22–33)	25 (20–30)	40 (36–41)	19 (18–20)		19 (18–20)
SP	30 (25–34)	24 (23–26)	41 (38–49)	20 (18–20)		25 (20–38)
TO	27 (22–36)	27 (26–30)	45 (38–53)	18 (18–19)		18 (18–21)
Median	30 (23–35)	26 (22–28)	40 (38–48)	18 (18–20)	22 (21–27)	20 (18–30)

AC, Acre. BA, Bahia. GO, Goiás. MG, Minas Gerais. MS, Mato Grosso do Sul. MT, Mato Grosso. PA, Pará. RO, Rondônia. SP, São Paulo, TO, Tocantins. Mixed, more than one truck type per freight order. Trailer36, trailers with an average load capacity of 36 animals. Trailer54, trailers with an average load capacity of 54 animals. Truck18, rigid trucks with an average load capacity of 18 animals. Truck36, rigid trucks with an average load capacity of 36 animals.

Supplementary Table S2. Median distance (km) and 25th and 75th percentiles travelled from the farm to the slaughterhouse by truck class and state

State	Truck class					Median
	Mixed	Trailer36	Trailer54	Truck18	Truck36	
AC	106 (84–128)		154 (100–216)	106 (67–220)		106 (67–220)
BA	300 (80–510)	380 (84–522)	255 (80–430)	220 (105–440)		220 (102–440)
GO	160 (90–280)	200 (100–320)	210 (100–325)	210 (120–325)	127 (60–200)	198 (100–310)
MG	132 (85–222)	121 (78–200)	260 (119–410)	104 (74–166)	90 (64–123)	113 (78–190)
MS	200 (105–300)	176 (102–280)	225 (120–328)	190 (110–280)	180 (110–290)	200 (110–295)
MT	136 (80–230)	130 (70–180)	156 (90–250)	112 (65–185)	115 (60–214)	127 (72–210)
PA	160 (110–230)	125 (90–217)	180 (105–215)	160 (110–245)		160 (110–240)
RO	197 (150–308)	161 (129–278)	250 (160–347)	195 (97–380)		197 (103–375)
SP	124 (33–220)	236 (125–408)	220 (108–383)	170 (115–325)		180 (82–314)
TO	180 (140–320)	309 (180–509)	295 (217–460)	165 (120–225)		175 (125–260)
Median	160 (89–260)	146 (89–256)	196 (105–309)	150 (85–260)	156 (93–255)	160 (89–267)

AC, Acre. BA, Bahia. GO, Goiás. MG, Minas Gerais. MS, Mato Grosso do Sul. MT, Mato Grosso. PA, Pará. RO, Rondônia. SP, São Paulo, TO, Tocantins. Mixed, more than one truck type per freight order. Trailer36, trailers with an average load capacity of 36 animals. Trailer54, trailers with an average load capacity of 54 animals. Truck18, rigid trucks with an average load capacity of 18 animals. Truck36, rigid trucks with an average load capacity of 36 animals.

Supplementary Table S3. Median carcass weight of females (kg) and the 25th and 75th percentiles by truck class and originating state

State	Truck class					Median
	Mixed	Trailer36	Trailer54	Truck18	Truck36	
AC	239 (219–245)			215 (193–239)		215 (193–239)
BA	216 (205–238)	207 (195–225)	199 (186–211)	218 (199–239)		216 (197–237)
GO	244 (212–273)	223 (205–246)	225 (206–256)	220 (204–241)	219 (204–236)	224 (206–251)
MG	241 (208–276)	226 (203–257)	234 (208–260)	222 (204–246)	218 (203–246)	225 (204–252)
MS	223 (206–246)	225 (207–247)	223 (207–244)	221 (204–241)	224 (207–246)	222 (205–243)
MT	227 (208–254)	218 (204–238)	224 (207–245)	219 (204–238)	212 (198–235)	220 (205–240)
PA	225 (206–243)	217 (200–237)	208 (191–224)	212 (196–234)		213 (197–234)
RO	214 (190–243)	194 (182–215)	201 (182–225)	197 (183–218)		198 (183–219)
SP	233 (210–261)	225 (205–249)	223 (204–248)	221 (204–244)		223 (205–247)
TO	210 (193–236)	218 (198–244)	210 (193–236)	203 (188–221)		205 (189–225)
Median	228 (207–255)	222 (204–246)	223 (205–246)	214 (196–235)	222 (205–244)	217 (199–240)

AC, Acre. BA, Bahia. GO, Goiás. MG, Minas Gerais. MS, Mato Grosso do Sul. MT, Mato Grosso. PA, Pará. RO, Rondônia. SP, São Paulo, TO, Tocantins. Mixed, more than one truck type per freight order. Trailer36, trailers with an average load capacity of 36 animals. Trailer54, trailers with an average load capacity of 54 animals. Truck18, rigid trucks with an average load capacity of 18 animals. Truck36, rigid trucks with an average load capacity of 36 animals.

Supplementary Table S4. Median carcass weight of males (kg) at the 25th and 75th percentiles by truck class and originating state

State	Truck class					Median
	Mixed	Trailer36	Trailer54	Truck18	Truck36	
AC	282 (269–298)		273 (268–293)	272 (259–286)		272 (259–286)
BA	290 (274–304)	278 (264–291)	287 (273–300)	284 (266–300)		284 (268–300)
GO	308 (289–328)	296 (278–318)	298 (280–316)	290 (272–308)	297 (277–311)	300 (281–320)
MG	298 (281–315)	290 (272–308)	297 (277–311)	286 (269–306)	288 (258–315)	290 (273–309)
MS	295 (278–312)	291 (274–312)	292 (276–309)	290 (274–310)	291 (272–309)	291 (275–310)
MT	315 (296–335)	304 (285–325)	309 (291–330)	299 (280–319)	285 (276–342)	306 (287–327)
PA	296 (283–311)	291 (277–308)	288 (276–299)	289 (276–303)		289 (277–304)
RO	296 (282–312)	278 (265–301)	286 (270–303)	284 (267–301)		285 (268–303)
SP	300 (282–319)	286 (268–307)	294 (276–312)	282 (265–303)		292 (274–312)
TO	291 (281–307)	289 (275–303)	290 (278–308)	289 (276–304)		289 (277–305)
Median	303 (285–323)	293 (275–312)	298 (280–317)	288 (272–306)	291 (272–309)	293 (276–312)

AC, Acre. BA, Bahia. GO, Goiás. MG, Minas Gerais. MS, Mato Grosso do Sul. MT, Mato Grosso. PA, Pará. RO, Rondônia. SP, São Paulo, TO, Tocantins. Mixed, more than one truck type per freight order. Trailer36, trailers with an average load capacity of 36 animals. Trailer54, trailers with an average load capacity of 54 animals. Truck18, rigid trucks with an average load capacity of 18 animals. Truck36, rigid trucks with an average load capacity of 36 animals.

Supplementary Table S5. Median and 25th and 75th percentiles of linear space (meters) occupied per animal during transport across different truck classes

Sex	Truck class				Median
	Trailer36	Trailer54	Truck18	Truck36	
Female	0.54 (0.50–0.59)	0.60 (0.54–0.67)	0.48 (0.48–0.54)	0.57 (0.45–0.57)	0.51 (0.48–0.55)
Male	0.55 (0.54–0.59)	0.60 (0.57–0.65)	0.54 (0.48–0.54)	0.57 (0.46–0.60)	0.54 (0.52–0.59)

Trailer36, trailers with an average load capacity of 36 animals. Trailer54, trailers with an average load capacity of 54 animals. Truck18, rigid trucks with an average load capacity of 18 animals. Truck36, rigid trucks with an average load capacity of 36 animals.

Supplementary Table S6. Median estimates and 25th and 75th percentiles of CO_2 equivalent emissions (CO_2e) per animal and per ton of carcass, based on the round–trip distance for each freight order and emissions per kilometer travelled

	Per round–trip distance (kg)		Per kilometer travelled (g)	
Truck class	Animal	Carcass ton	Animal	Carcass ton
Truck18	13.99 (7.8–23.8)	56.89 (31.6–98)	46.49 (43.7–48.6)	187.2 (164.6–218)
Truck36	10.19 (6.2–16.9)	40.89 (24.2–68.2)	35.64 (29–37.3)	133.01 (118.5–155.7)
Trailer36	9.00 (5.3–15.7)	35.66 (20.5–62.5)	30.15 (28–35.6)	120.59 (101.4–141.4)
Trailer54	10.32 (5.6–16.4)	37.77 (20.5–62.5)	27.34 (22.8–28.8)	96.83 (82.9–117.7)
Median	12.54 (7.0–21.4)	50.13 (27.7–86.5)	43.7 (32.7–48.6)	170.35 (131.9–206.8)

Trailer36, trailers with an average load capacity of 36 animals. Trailer54, trailers with an average load capacity of 54 animals. Truck18, rigid trucks with an average load capacity of 18 animals. Truck36, rigid trucks with an average load capacity of 36 animals.

Supplementary Table S7. Median estimates and 25th and 75th percentiles of CO_2 equivalent (CO_2e, kg) emissions per animal and per ton of carcass by state, considering transport distance (round trip)

State	Animal	Carcass ton
Acre	9.88 (6.16–20.06)	38.67 (24.54–80.34)
Bahia	16.70 (7.93–36.71)	63.92 (29.15–132.72)
Goiás	13.60 (7.00–22.34)	51.65 (26.27–89.93)
Minas Gerais	8.74 (5.83–15.05)	34.40 (22.31–58.98)
Mato Grosso do Sul	13.84 (7.96–20.98)	54.44 (31.15–85.04)
Mato Grosso	9.72 (5.54–15.95)	38.63 (21.86–66.22)
Pará	14.57 (9.71–22.34)	55.95 (36.74–83.46)
Rondônia	17.31 (8.57–34.53)	74.73 (37.38–153.93)
São Paulo	13.60 (7.64–23.81)	52.96 (28.94–95.28)
Tocantins	15.83 (11.40–21.62)	57.39 (40.22–82.78)
Median	12.54 (7.00–21.37)	50.13 (27.65–86.48)
